# Mutations in Epigenetic Regulation Genes Are a Major Cause of Overgrowth with Intellectual Disability

**DOI:** 10.1016/j.ajhg.2017.03.010

**Published:** 2017-05-04

**Authors:** Katrina Tatton-Brown, Chey Loveday, Shawn Yost, Matthew Clarke, Emma Ramsay, Anna Zachariou, Anna Elliott, Harriet Wylie, Anna Ardissone, Olaf Rittinger, Fiona Stewart, I. Karen Temple, Trevor Cole, Childhood Overgrowth Collaboration, Shazia Mahamdallie, Sheila Seal, Elise Ruark, Nazneen Rahman

**Affiliations:** 1 Division of Genetics and Epidemiology, Institute of Cancer Research, 15 Cotswold Road, London SM2 5NG, UK; 2 South West Thames Regional Genetics Service, St George’s University Hospitals NHS Foundation Trust, London SW17 0QT, UK; 3 Child Neurology Unit, Foundation IRCCS C Besta Neurological Institute, Milan 20133, Italy; 4 Landeskrankenanstalten Salzburg, Kinderklinik Department of Pediatrics, Klinische Genetik, Salzburg 5020, Austria; 5 Northern Ireland Regional Genetics Service, Belfast City Hospital, Belfast BT9 7AB, Northern Ireland; 6 Human Development and Health Academic Unit, Faculty of Medicine, University of Southampton, Southampton SO17 1BJ, UK; 7 Wessex Clinical Genetics Service, University Hospital Southampton NHS Trust, Southampton SO16 6YD, UK; 8 West Midlands Regional Genetics Service, Birmingham Women’s Hospital NHS Foundation Trust and University of Birmingham, Birmingham Health Partners, Birmingham B15 2TG, UK; 9 Cancer Genetics Unit, Royal Marsden NHS Foundation Trust, London SW3 6JJ, UK

**Keywords:** overgrowth syndrome, epigenetic regulation, exome sequencing, intellectual disability, sotos syndrome, weaver syndrome, HIST1H1E, NSD1, EZH2

## Abstract

To explore the genetic architecture of human overgrowth syndromes and human growth control, we performed experimental and bioinformatic analyses of 710 individuals with overgrowth (height and/or head circumference ≥+2 SD) and intellectual disability (OGID). We identified a causal mutation in 1 of 14 genes in 50% (353/710). This includes *HIST1H1E*, encoding histone H1.4, which has not been associated with a developmental disorder previously. The pathogenic *HIST1H1E* mutations are predicted to result in a product that is less effective in neutralizing negatively charged linker DNA because it has a reduced net charge, and in DNA binding and protein-protein interactions because key residues are truncated. Functional network analyses demonstrated that epigenetic regulation is a prominent biological process dysregulated in individuals with OGID. Mutations in six epigenetic regulation genes—*NSD1*, *EZH2*, *DNMT3A*, *CHD8*, *HIST1H1E*, and *EED*—accounted for 44% of individuals (311/710). There was significant overlap between the 14 genes involved in OGID and 611 genes in regions identified in GWASs to be associated with height (p = 6.84 × 10^−8^), suggesting that a common variation impacting function of genes involved in OGID influences height at a population level. Increased cellular growth is a hallmark of cancer and there was striking overlap between the genes involved in OGID and 260 somatically mutated cancer driver genes (p = 1.75 × 10^−14^). However, the mutation spectra of genes involved in OGID and cancer differ, suggesting complex genotype-phenotype relationships. These data reveal insights into the genetic control of human growth and demonstrate that exome sequencing in OGID has a high diagnostic yield.

## Introduction

Human growth control, at the organismal and cellular level, is a complex process essential for health and dysregulated in many developmental disorders and cancers. The mechanistic control of cell size and proliferation has been studied, by diverse approaches, in many different species.^1^, ^2^ However, the control of overall size of an organism has been relatively understudied and is still poorly understood. The study of human growth disorders therefore not only improves diagnosis and management of human disease, it also offers an opportunity to enhance knowledge about the fundamental processes governing control of human size.

Human overgrowth syndromes are a nebulous group of conditions defined as having height and/or head circumference ≥2 SD above the mean, together with additional phenotypic abnormalities, the most common of which is intellectual disability.^3^ Overgrowth syndromes usually occur sporadically within a family and can be caused by several different mechanisms, including gene mutations, imprinting disruption, and chromosome dosage abnormalities.^3^, ^4^

Single-gene disorders associated with overgrowth and intellectual disability (OGID) are well recognized; Sotos syndrome (MIM: 117550) and Weaver syndrome (MIM: 277590) are prototypic examples, due to *NSD1* (MIM: 606681) and *EZH2* (MIM: 601573) mutations, respectively (see GeneReviews by Tatton-Brown et al. in Web Resources).^5^ OGID syndromes have been increasingly identified over the last decade.^3^, ^4^ The advent of next-generation sequencing has been the foremost reason for this progress and has allowed elucidation of the genetic causes of clinically established syndromes and the delineation of new syndromes.^5^, ^6^, ^7^, ^8^, ^9^, ^10^, ^11^, ^12^

Despite these advances, many individuals with OGID remain without a genetic diagnosis. In addition, the relative contribution of the different genes to OGID is unknown. To better characterize the genetic landscape of OGID, we have here studied 710 affected individuals including 323 parent-proband trios (Table S1).

## Subjects and Methods

### Subjects

We recruited participants through the Childhood Overgrowth (COG) Study, which began recruitment in 2005, approved by the London Multicenter Ethics Committee (05/MRE02/17). Informed consent was obtained from all participants and/or parents, as appropriate. Individuals were eligible for this study if they had height and/or head circumference at least two standard deviations above the mean (≥+2 SD, UK90 growth data)^13^ at some point in childhood, together with intellectual disability. We have termed this condition OGID (overgrowth + intellectual disability). Overgrowth phenotypes that are not associated with intellectual disability, such as Beckwith Wiedemann syndome (MIM: 130650) or Marfan syndrome (MIM: 154700), were not included. Regional or asymmetric overgrowth phenotypes (e.g., hemihypertrophy) in the absence of increased height or head circumference were not included.

710 individuals with OGID were included. 97% (693) were recruited to the study from clinical genetics departments. For 323 individuals, samples from both parents were also available and included. 205 probands had both height and head circumference ≥+2 SD, termed “head+height” in Table S1. 138 had height ≥+2 SD with OFC <2 SD, termed “height only” and 109 had OFC ≥+2 SD and height <2 SD, termed “head only.” For the remaining 258 individuals, the child was recruited to the study because they had overgrowth, but measurements for both height and head were not provided. The overgrowth category is termed “unspecified” for these case subjects in Table S1. Intellectual disability was classified by the referring clinician as severe (77 case subjects), moderate (228 case subjects), or mild (229 case subjects). The referrer did not state the severity of the OGID for 176 individuals (termed “unspecified” in Table S1).

### Control Data

We used the Exome Aggregation Consortium (ExAC) data v.3 accessed on 13/11/2015 (excluding the TCGA samples)^14^ and the ICR1000 UK exome series^15^ as reference data. We generated and analyzed the ICR1000 UK exome series data using the same sequencing and analysis pipeline described for the OGID samples.

### Targeted Gene Analyses

We previously reported mutations in *NSD1*, *EZH2*, *DNMT3A* (MIM: 602769), and *PPP2R5D* (MIM: 601646) in 198 case subjects. The relevant references are in Table S1. Intragenic mutations in these genes were detected with Sanger sequencing. *NSD1* is unusual among the 14 OGID genes included in this study in being prone to deletion by a 2 Mb 5q35 microdeletion, mediated by flanking low-copy repeats.^16^ We used MLPA to identify 5q35 microdeletions encompassing *NSD1*.^17^ *NSD1* MLPA is also capable of detecting exon CNVs that account for ∼5% of *NSD1* mutations.^17^ Microdeletions and exon CNVs in the other genes were not sought, but are unlikely to be a major contributor because the surrounding sequence architecture and/or mechanism of pathogenicity make it much less likely that such events will cause OGID.

### Exome Sequencing

We performed exome sequencing in all probands in whom no mutation had been identified by targeted gene analyses and in parental samples where available. We performed exome sequencing using the Nextera Rapid Capture Exome Kit (Illumina). We prepared libraries from 50 ng genomic DNA using the Nextera DNA Sample Preparation Kit (Illumina). On average 33M reads mapped to the pulldown and 86% of targeted bases had ≥15× coverage. The captured libraries were PCR amplified using the supplied paired-end PCR primers. Exome sequencing in 57 samples was performed before the Nextera Exome Kit was available using the TruSeq Exome Enrichment Kit, which includes the 14 genes involved in OGID. When converting our exome pipeline from TruSeq to Nextera, we undertook in-house evaluation and validation to ensure that the performance was equivalent. Sequencing was performed on an llumina HiSeq 2000 or HiSeq 2500 (high output mode) using v3 chemistry and generating 2 × 101 bp reads.

### Variant Calling

We used the OpEx v1.0 pipeline to perform variant calling.^18^ We converted raw data to FASTQs using CASAVA v.1.8.2 with default settings. The OpEx v1.0 pipeline uses Stampy^19^ to map to the human reference genome, Picard to flag duplicates, Platypus^20^ to call variants, and CAVA^21^ to provide consistent annotation of variants with the HGVS-compliant CSN (Clinical Sequencing Notation) standard v1.0.^21^ The transcript information for variant annotation for the 14 relevant genes are given in Table 1.

### Variant Prioritization and Validation

We excluded variants with MAF > 0.5% in either the Exome Aggregation Consortium (ExAC) and/or the ICR1000 UK exome series. For the de novo analyses, we identified and validated any high-quality (as defined by OpEx^18^) variant in the child that was not present in either parent. We evaluated and validated all rare variants identified in the 14 genes.

We confirmed all small variants in Table S1 that were called in exomes via Sanger sequencing of M13-tagged PCR products generated from genomic DNA. We performed PCR using the QIAGEN Multiplex PCR Kit according to the manufacturer’s instructions. We sequenced PCR products using M13 sequencing primers, the BigDye Terminator Cycle Sequencing Kit, and an ABI 3730 Genetic Analyzer (Applied Biosystems). We analyzed sequences using Mutation Surveyor software v.3.20 (SoftGenetics) and verified the outputs by manual inspection by two individuals, independently.

### Pathogenic Mutation Determination

Apart from *HIST1H1E* (MIM: 142220), we considered a variant in the other 13 genes to be pathogenic if it fulfilled one or more of the following criteria. (1) It was a de novo mutation in a gene for which such de novo mutations were already proven to cause OGID. (2) The inheritance was unknown, because parental samples were unavailable, but it had been previously identified as a pathogenic de novo mutation in OGID. (3) It was a protein-truncating variant ([PTV] frameshifting indels, stop-gain, or essential splice-site variants) in a gene in which truncating mutations have been proven to be pathogenic. (4) There was clear evidence from the literature that it was pathogenic. The evidence for *HIST1H1E* mutations being pathogenic is provided in the Results.

### *HIST1H1E* Statistical Analyses

We used the methods described in the DDD study^22^ to calculate the probability of identifying four de novo frameshift mutations in *HIST1H1E* using the gene-specific mutation rates from Samocha et al.^23^ The frameshift mutation rate in *HIST1H1E* (4.18 × 10^−7^) was multiplied by twice the number of case subjects in this study (710) in order to get the expected number of frameshift mutations. We calculated the probability of observing four or more de novo frameshift mutations in *HIST1H1E* given the expected number of frameshift mutations via the ppois function in R.

We modeled the significance of mutation clustering in *HIST1H1E* under a binomial distribution where the probability of observing a mutation in a 12 bp region, which comprises 1.8% of the coding sequence, was 0.018.

### Protein Net Charge Calculation

We obtained wild-type *HIST1H1E* cDNA (frame 1) sequence from Ensembl (ENST00000304218.5). We generated the *HIST1H1E* cDNA sequences edited with OGID mutations (frame 2). We used the variant c.430delG to generate the other possible alternative reading frame in *HIST1H1E* (frame 3). We translated the cDNA sequences using the Translate Tool at ExPASy. We calculated the net charge of the carboxy-terminal domain, from p.Lys110 onward, at neutral pH using the Peptide Property Calculator at the Innovagen website.

### Functional Network Analyses

We performed functional enrichment analysis using g:Profiler (v.r1665_e85_eg32).^24^ We used the 14 genes in Table 1 as our query set. We looked for enrichment among Gene Ontology molecular function terms and KEGG pathway gene sets, requiring the size of the functional category to be between 5 and 500 genes and using the Benjamini-Hochberg false discovery rate as the significance threshold. The FDR q values presented are the Benjamini-Hochberg critical values.

### Phenotypic Analyses

We tested for significant difference in the diagnostic yields between different phenotypic groups using the prop.test function in R. We calculated the significance of association between an individual having macrocephaly and their mutation status (either a mutation in a PI3K/AKT pathway gene or a mutation in an epigenetic regulation gene) using a Fisher’s exact test, which we implemented with the fisher.test function in R. We calculated the significance of association between an individual having macrocephaly in the absence of increased height and their mutation status, and the significance of association between an individual having increased height in the absence of macrocephaly and their mutation status in the same way. We tested for significant difference in the proportion of individuals with mild intellectual disability for those with a mutation in a PI3K/AKT pathway OGID gene and those with a mutation in an epigenetic regulation OGID gene using the prop.test function in R.

### Height GWAS Gene and Cancer Driver Gene Comparisons

We obtained the list of 611 genes located in regions associated with human height through GWASs from Table S1 of Wood et al.^25^ We obtained a list of 260 somatically mutated cancer genes from Table S2 of Lawrence et al.^26^ and the somatic mutations from the tumor portal website.

We calculated the probability of seeing the observed overlap of the OGID gene set with the GWAS gene set under a hypergeometric probability distribution assuming a total hypothetical size of 20,000 protein-coding genes in the exome using the phyper function in R. We calculated the probability of seeing the observed overlap of OGID gene set with the cancer driver gene set in the same way.

## Results

### Contribution of Gene Mutations to OGID

**Figure 1:**
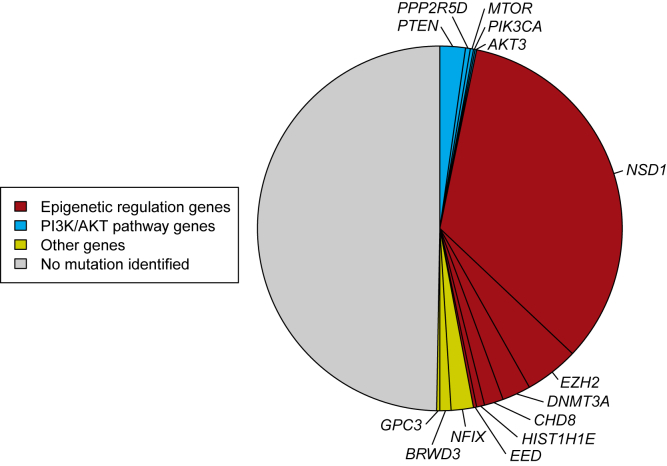
Causal Mutation Identified in 50% of OGID Probands Proportion of pathogenic mutations identified in 710 individuals with OGID. Epigenetic regulation genes (red), including *NSD1* which is the predominant gene, constitute the major gene set. PI3K/AKT pathway genes (blue) also significantly contribute to OGID.

Using exome or targeted gene analyses, we identified a pathogenic mutation in one of 14 genes in 357 individuals with OGID, giving a diagnostic yield of 50% (Figure 1). By far the most common cause was a mutation in *NSD1* (240 cases, 34%), followed by *EZH2* (34, 4.8%), *DNMT3A* (18, 2.5%), *PTEN* (MIM: 601728) (16, 2.3%), *NFIX* (MIM: 164005) (14, 2.0%), *CHD8* (MIM: 610528) (12, 1.7%), *BRWD3* (MIM: 300553) (7, 1.0%), *HIST1H1E* (5, 0.7%), *PPP2R5D* (3, 0.4%), (2 cases each) *EED* (MIM: 605984), *GPC3* (MIM: 300037), and *MTOR* (MIM: 601231), and (1 case each) *AKT3* (MIM: 611223) and *PIK3CA* (MIM: 171834) (Table S1). Among the 323 parent-proband trios, we identified a cause in 191 (59%) of which 179 were de novo mutations and 12 were inherited.

Our data allow confirmation that *EED* mutations cause OGID. Two case reports of individuals with a characteristic phenotype that includes overgrowth have been published.^10^, ^27^ We here present two additional cases with a de novo *EED* mutation. The individuals have the same facial phenotype to each other and to previously reported case subjects, with long, narrow palpebral fissures, telecanthus, and retrognathia. Notably, EED is a direct binding partner of EZH2,^28^ which has an established role in causing OGID.^29^ Some role in overgrowth was either known, or has been proposed, for the remainder of these, apart from *HIST1H1E* (see GeneReviews by Eng in Web Resources).^6^, ^9^, ^10^, ^12^, ^29^, ^30^, ^31^, ^32^, ^33^, ^34^, ^35^

### *HIST1H1E* Mutations Cause OGID

**Figure 2:**
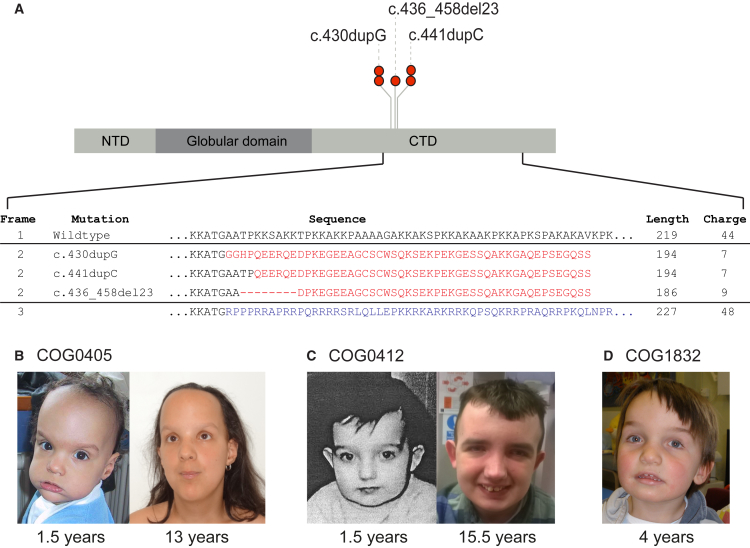
*HIST1H1E* Mutations Cause OGID (A) *HIST1H1E* mutations cluster within 12 bp region in the carboxy-terminal domain (CTD) and have a similar predicted impact on protein function. The three different frameshift mutations generate the same open reading frame (frame 2), which is predicted to reduce the length and net charge (at pH 7) of the CTD compared to the wild-type (frame 1). The other possible alternate reading frame (frame 3) increases the protein length and net charge. Abbreviations: CTD, carboxy-terminal domain; NTD, amino-terminal domain. (B–D) Facial images of three individuals with *HIST1H1E* mutations showing full cheeks and high hairline.

We present here data showing that certain *HIST1H1E* mutations cause OGID. Through exome sequencing we identified five unrelated probands—COG0405, COG0412, COG0552, COG1739, and COG1832—with heterozygous *HIST1H1E* protein truncating variants (PTVs) (Figure 2, Tables 1 and S1). In four probands the PTV had arisen de novo. Parental samples were not available for the fifth child, but she carried the same mutation as one of the children with a de novo mutation. The detection of four de novo *HIST1H1E* mutations in 710 individuals is highly unlikely to have occurred by chance, as determined from gene-specific de novo mutation rates (p = 5.17 × 10^−15^). None of the mutations are present in the ExAC dataset, nor in 11,677 exomes analyzed in-house with similar pipelines. These results strongly support *HIST1H1E* mutations as a cause of OGID.

*HIST1H1E* encodes histone H1.4. In humans, H1.4 is one of 11 H1 linker histones that mediate the formation of higher-order chromatin structures and regulate the accessibility of regulatory proteins, chromatin remodelling factors, and histone-modifying enzymes to their target sites.^36^, ^37^ The five mutations we identified cluster significantly (p = 2.0 × 10^−9^) to a 12-bp region in the carboxy-terminal domain (CTD) that is involved in chromatin binding and protein-protein interactions (Figure 2A).^36^ PTVs in the intronless histones have been shown to evade nonsense-mediated mRNA decay.^38^ Thus the OGID-causing mutations are predicted to generate a truncated product.

The CTD of linker histones regulate higher-order chromatin structure through neutralization of negatively charged linker DNA.^36^ The pathogenic *HIST1H1E* mutations all result in the same shift in the reading frame and are predicted to generate similar truncated proteins, with a reduced net charge of 7–9 (compared to 44 for the wild-type protein) (Figure 2A). The mutant protein is thus likely to be less effective in neutralizing negatively charged linker DNA. Moreover, the truncation of the C-terminus likely impedes DNA binding and protein-protein interactions. It is also noteworthy that the other possible alteration in reading frame would reduce neither the net charge nor the length of the protein (Figure 2A). Taken together, these data suggest that specific *HIST1H1E* mutations, restricted in position and type, cause human overgrowth.

### *HIST1H1E* Clinical Phenotype

Individuals with *HIST1H1E* mutations had similar facial appearance in childhood with full cheeks, high hairline, and telecanthus (Figures 2B–2D). Height, head circumference, and degree of intellectual disability were variable, as were the additional clinical features. It is currently unclear whether these additional features are *HIST1H1E* associations or coincidental findings. Individual case descriptions are below.

COG0405, a female individual, was born at term with a weight of 3.58 kg (+0.1 SD) and a length of 53 cm (+1.5 SD). She was floppy in the neonatal period. A brain MRI scan at 4 months demonstrated mild ventricular dilatation but no other abnormalities. Her bone age at chronological age of 7 months was advanced to 18–24 months. By 19 months, her length was 87 cm (+2.0 SD) with a weight of 13.4 kg (+1.8 SD) and she had developed a strabismus. At 13 years of age, the individual was noted to have normal growth with a height of 150.8 cm (−0.6 SD), a head circumference of 55.8 cm (−0.5 SD), and a weight of 48.85 kg (+0.4 SD). She has developed a severe kyphoscoliosis for which she required surgery and has a mild intellectual disability.

COG0412, a male individual, was born at 1 week after term following an uncomplicated pregnancy and delivery. He weighed 4.75 kg (+2.4 SD). In the neonatal period he was noted to be floppy; he had poor feeding and undescended testes. At 1.5 years he was very tall at 105 cm (+8.3 SD) with a weight of 18.8 kg (+4.6 SD) and a head circumference of 52.5 cm (+2.6 SD). He was reported to have multiple nevi and redundant skin on the palms of his hands. He had a moderate intellectual disability and no behavioral issues at that time. When he was reviewed at 15.5 years, he was no longer tall with a height of 166.5 cm (−0.6 SD). His head circumference was 58.7 cm (+1.4 SD). By this age he had developed an anxiety disorder that was refractory to medical treatment. He had also developed phobias. In addition, he had major dental problems with crumbling teeth and he had dry, flaky nails.

COG0552, a female individual, was born at term with a weight of 4.79 kg (+2.5 SD) and length of 57 cm (+3.6 SD). She was floppy in the neonatal period with poor feeding. She developed no new medical problems in childhood. At the age of 4.2 years she was reported to be delayed in her development. She had a height of 108 cm (+1.2 SD), head circumference of 55 cm (+3.2 SD), and weight of 24 kg (+2.7 SD).

COG1739, a female individual, was initially thought clinically to have Weaver syndrome. She was born at 37 weeks after an uncomplicated pregnancy and labor with a weight of 3.25 kg (+0.8 SD), length of 49 cm (+0.7 SD), and head circumference of 37 cm (+3.3 SD). She was hypoglycemic and hypertonic in the neonatal period, and was also noted to have camptodactyly. At 1.9 years she was diagnosed with a moderate intellectual disability and had a height of 85 cm (mean), head circumference of 51 cm (+1.8 SD), and weight of 12 kg (−0.3 SD).

COG1832, a male individual, was born at 1 week after term weighing 3.74 kg (+0.4 SD). The pregnancy had been complicated by exposure to chicken pox. At birth, COG1832 was noted to have talipes equinovarus and later in the neonatal period was diagnosed with delayed visual maturation. A brain MRI scan showed a slender corpus callosum and unusual ventricular outline, possibly indicative of a periventricular leukomalacia. At 8.5 years, height was 133.2 cm (+0.5 SD) with a weight of 33 kg (+1.2 SD). The head circumference at 6.3 years was 59 cm (+3.7 SD). He has limited speech but with verbal comprehension markedly ahead of this ability to express himself. He has left amblyopia and astigmatism. His hearing is normal. He suffers from constipation. At times his behavior is challenging.

### Functional Network Analyses

To investigate the biological processes abrogated by OGID pathogenic mutations, we performed functional enrichment analysis using the GO molecular function terms and KEGG pathway gene sets in g:Profiler.^24^ The chromatin binding (FDR q value = 1.58 × 10^−6^) and PI3K/AKT signaling pathway (FDR q value = 6.80 × 10^−5^) gene sets were significantly enriched.

**Figure 3:**
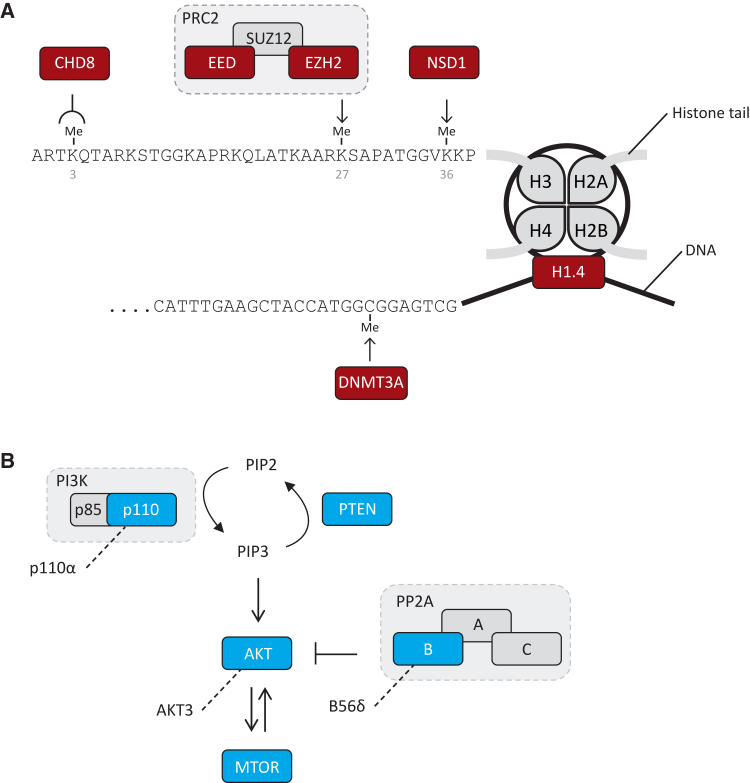
Schematic of Key Biological Processes Impacted in OGID (A) Epigenetic regulation. NSD1, EED, and EZH2 directly methylate specific histone tail lysine residues. DNMT3A is a de novo DNA methyltransferase and CHD8 is a chromatin remodeling complex protein that binds methylated lysine 4 of histone H3. H1.4 (encoded by *HIST1H1E*) stabilizes higher-order chromatin structures. (B) All OGID mutations are predicted to lead to reduced function PI3K/AKT pathway. The PI3K/AKT pathway positively regulates growth. AKT3, MTOR, and p110α (encoded by *PIK3CA*) are pathway activators. PTEN and B56δ (encoded by *PPP2R5D*) are pathway suppressors. OGID mutations in *AKT3*, *MTOR*, and *PIK3CA* are activating, whereas OGID mutations in *PTEN* and *PPP2R5D* are inactivating.

Six genes—*NSD1*, *EZH2*, *DNMT3A*, *EED*, *CHD8*, and *HIST1H1E*—were in the chromatin binding gene set. All encode proteins involved in epigenetic regulation (Figure 3A). NSD1 is a histone methyltransferase that catalyzes methylation of H3K36, and to lesser extent H4K20, and is primarily associated with transcriptional activation.^39^ EZH2 and EED are key components of the polycomb repressive complex 2 (PRC2), which catalyzes methylation of H3K27, resulting in transcriptional repression of target genes.^28^ DNMT3A is a DNA methyltransferase crucial for the establishment of new methylation marks during early embryogenesis and the sex-dependent methylation of imprinted genes.^40^, ^41^ *CHD8* encodes an ATP-dependent chromatin remodeler that binds to methylated H3K4, a key histone modification at active promoters.^35^ As noted above, H1.4 binds to linker DNA between nucleosomes and has key roles in chromatin compaction and regulation of gene expression.^37^ Together, mutations in these six genes accounted for 311 (44%) of our series. Disruption of epigenetic regulation is therefore a prominent molecular mechanism underlying OGID (Figure 1).

Five of the genes—*PTEN*, *AKT3*, *PIK3CA* (which encodes p110α, the catalytic domain of the heterodimeric PI3K lipid kinase), *MTOR*, and *PPP2R5D* (which encodes B56δ a regulatory subunit of the heterotrimeric PP2A protein phosphatase)—are in the PI3K/AKT pathway, which plays a key role in the regulation of growth (Figure 3B). Activation of the PI3K/AKT pathway results in cellular growth promotion through increased cell metabolism, cell survival, cell turnover, and protein synthesis.^42^ Together mutations in these genes made only a minor contribution to our OGID series (23 case subjects, 3.2%). In part this is because individuals with mutations in these genes are more often diagnosed with other conditions, such as Cowden syndrome (MIM: 158350), megalencephaly-capillary malformation syndrome (MIM: 602501), or regional overgrowth (see GeneReviews by Eng in Web Resources).^34^

The remaining three genes—*NFIX*, *GPC3*, and *BRWD3*—encode a transcription factor, a proteoglycan, and a bromodomain-containing protein, respectively^6^, ^31^, ^32^ (23 case subjects, 3.2%). There is currently no clear functional link between these genes and the other genes we report here. However, it is possible that *BRWD3* mutations also cause overgrowth through epigenetic regulation dysfunction, as there are data suggesting it is involved in histone H3.3 regulation.^43^

### Phenotype Analyses

There was enrichment of mutations in individuals with both increased height and head circumference, compared to individuals in whom only one growth parameter was increased, as would be expected. Specifically the diagnostic yield in individuals with both macrocephaly and increased height was 59% (120/205), significantly higher than the diagnostic yields in individuals with only macrocephaly (43%, 47/109, p = 0.006) or only increased height (45%, 62/138, p = 0.009). There was no significant difference between the diagnostic yields in individuals with only macrocephaly and in those with only increased height (p = 0.146). There was also no significant difference between the diagnostic yield in individuals with unspecified growth parameters (50%, 130/258) and any other group.

**Figure 4:**
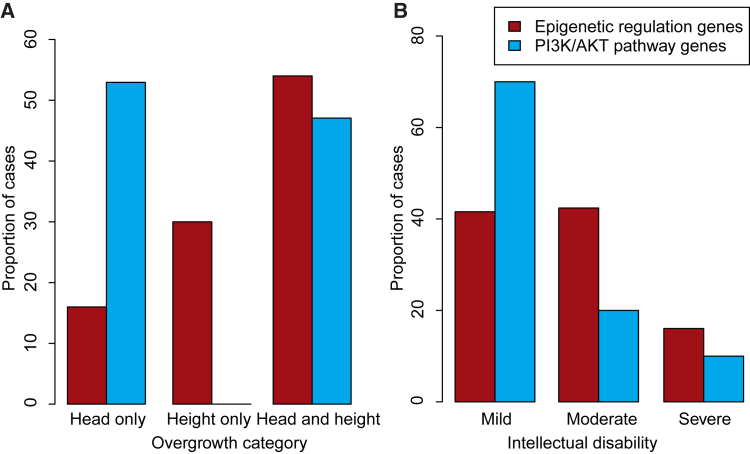
Phenotypic Differences between OGID due to Mutations in Epigenetic Regulation Genes Compared to PI3K/AKT Pathway Genes Comparison of the distribution of (A) overgrowth categories and (B) degree of intellectual disability in case subjects with epigenetic regulation gene mutations (red) compared with PI3K/AKT pathway gene mutations (blue).

To further explore the phenotypic spectrum of OGID, we compared the growth and intellectual disability severity of the individuals due to mutations in the epigenetic regulation genes and those involved in the PI3K/AKT pathway, using case subjects for which the relevant phenotypic information was available (217 individuals with complete growth data and 263 individuals with intellectual disability severity information) (Figure 4). Macrocephaly (i.e., head circumference ≥2 SD above the mean) occurred more frequently in individuals with PI3K/AKT pathway gene mutations; all 17 had macrocephaly, compared with 140/200 individuals with OGID due to epigenetic regulation gene mutations (p = 4.1 × 10^−3^; Figure 4A). Furthermore, 9/17 of the PI3K/AKT pathway case subjects had macrocephaly without increased height compared with 32/200 of the epigenetic regulation pathway cases (p = 1.0 × 10^−3^; Figure 4A). The remaining 60/200 had increased height without macrocephaly, a combination not present in OGID due to PI3K/AKT pathway gene mutations (p = 4.1 × 10^−3^; Figure 4A). Varying severity of intellectual disability was a feature of both groups, but mild intellectual disability was more common in OGID due to PI3K/AKT pathway gene mutations (14/20) than OGID due to epigenetic regulation gene mutations (101/243; p = 0.01) (Figure 4B).

The risk of childhood cancer is one the most controversial areas of OGID management. 8/710 OGID-affected individuals in this study developed cancer in childhood (Table S1). This includes 4/357 with an identified genetic cause, three of whom had an *EZH2* mutation. COG1724 developed neuroblastoma at 46 months, COG0285 developed T cell non-hodgkins lymphoma at 13 years, and COG1521 was diagnosed with both neuroblastoma and acute lymphoblastic leukemia at 13 months. The childhood cancer incidence for *EZH2* mutation carriers in this study was thus 9% (3/34). The remaining child had an *NSD1* microdeletion and T cell non-hodgkins lymphoma. This information will be useful in family discussions about childhood cancer risk, particularly in relation to surveillance strategies, which are generally of unproven benefit and can be associated with appreciable false positive rates.^44^

### Height GWAS Loci Comparative Analyses

We next explored the overlap between the 14 genes and 611 genes implicated through genome-wide association studies (GWASs) to be involved in the control of human height.^25^ There was significant overlap; six genes involved in OGID were also present in height GWAS regions (p = 6.8 × 10^−8^) (Figure S1). The overlap is primarily through the epigenetic regulation genes, all of which (except *EED*) were represented in height GWAS regions. Two separate intronic SNPs in each of *NSD1* and *DNMT3A* were independently associated with height in the GWAS and there were no other genes within the linkage disequilibrium (LD) blocks of association. This strongly suggests that NSD1 and DNMT3A functional impact underlie the height association in these regions (Figure S1). Single SNPs in intron 5 of *CHD8*, intron 9 of *MTOR*, 1 kb downstream of *HIST1H1E*, and 48 kb upstream of *EZH2* were also associated with height.^25^ For *HIST1H1E* and *EZH2*, there were no other genes in the LD block of association. For *MTOR* the variant associated with a *cis*-eQTL affecting *MTOR* expression, though the association was better accounted for by an upstream variant (rs2295080) in the *MTOR* promoter region that was in LD with the height SNP (LD r^2^ = 0.85).^25^ Although the causal SNPs and mechanisms of association are not fully elucidated, these data suggest that common variation in some genes involved in OGID also influence height at a population level.

### Cancer Somatic Driver Mutation Comparative Analyses

Dysregulated cellular growth is a hallmark of cancer, and certain human conditions are associated with both overgrowth and increased cancer risk (see GeneReviews by Eng in Web Resources).^45^ We therefore next sought to investigate the overlap between the 14 genes and 260 somatically mutated cancer driver genes reported by Lawrence et al.^26^ There was significant overlap; 8/14 genes involved in OGID were somatically mutated in a diverse range of cancers (*NSD1*, *EZH2*, *DNMT3A*, *PTEN*, *CHD8*, *HIST1H1E*, *MTOR*, *PIK3CA*; p = 1.7 × 10^−14^). For the PI3K/AKT pathway genes, the mutation spectra are similar in OGID and cancer.^34^ By contrast, for the epigenetic regulation genes, the mutation spectra in OGID and cancer have substantial, distinctive differences.

**Figure 5:**
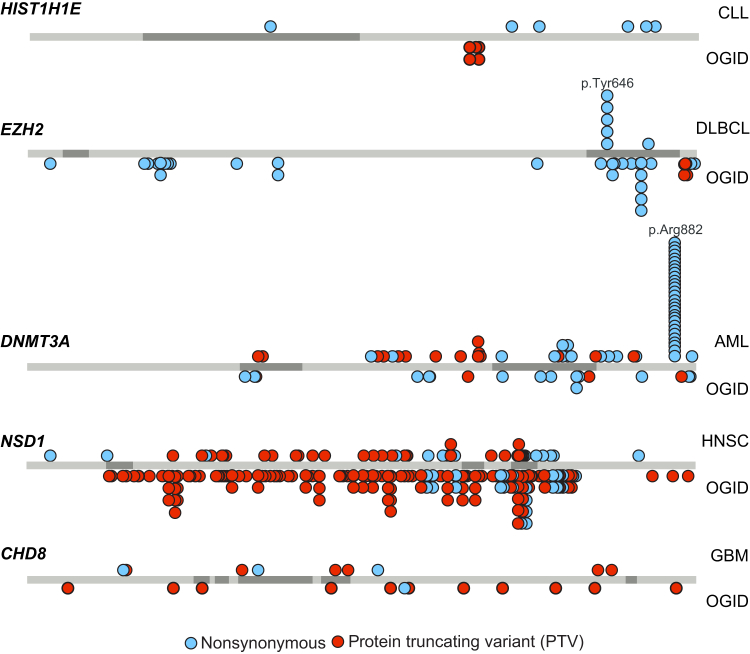
Mutations in Epigenetic Regulation Genes in OGID and Cancers Protein schematics showing the position of mutations in *HIST1H1E*, *EZH2*, *DNMT3A*, *NSD1*, and *CHD8* in OGID (below the gene) and specific cancers (above the gene). The somatic cancer driver mutations are from Lawrence et al.^26^ Abbreviations are as follows: AML, acute myeloid leukemia; CLL, chronic lymphocytic leukemia; DLBCL, diffuse large B-cell lymphoma; GBM, glioblastoma multiforme; HNSC, head and neck squamous cell carcinoma; OGID, overgrowth-intellectual disability.

Somatic mutations in *HIST1H1E*, *EZH2*, and *DNMT3A* occur in hematological malignancies.^26^, ^46^, ^47^, ^48^, ^49^, ^50^ *HIST1H1E* and *EZH2* mutations are each present in ∼20% of B cell lymphomas.^48^, ^49^ Somatic *HIST1H1E* mutations are nonsynonymous mutations throughout the gene and do not include the clustered PTVs that cause OGID (Figure 5). *EZH2* mutations in B cell lymphomas are often activating nonsynonymous mutations in the SET domain, the majority of which target a single amino acid, p.Tyr646.^48^ Nonsynonymous mutations at this residue have not been detected in OGID and are not present in ExAC, perhaps suggesting that germline *EZH2* mutations altering p.Tyr646 are not compatible with life (Figure 5). Inactivating *EZH2* mutations are present in myeloid malignancies and in T-ALL.^46^, ^47^, ^48^ A proportion of these latter mutations overlap with *EZH2* mutations in OGID.

*DNMT3A* is one of the most frequently mutated genes in AML and mutations also occur less frequently in other hematological malignancies.^26^, ^50^ The majority target a single residue, p.Arg882, with the remainder being nonsynonymous variants and PTVs scattered through the gene. Mutations at p.Arg882 have not thus far been reported in OGID (Figure 5). Protein modeling suggests that the somatic mutations primarily impact DNA binding, whereas the mutations in OGID are more likely to impact histone binding.^12^

Somatic *NSD1* mutations are seen in ∼10% of head and neck squamous cell carcinomas^26^, ^51^ and somatic *CHD8* mutations are present in ∼3% of glioblastoma multiforme (GBM).^26^ For these cancers the mutation pattern is similar to that observed in OGID, with PTVs being the most frequent mutation type (Figure 5).^30^ Interestingly, Lawrence et al. found *NSD1* and *CHD8* to each be significant in their pan-cancer analysis, present in 2% of cancers.^26^ However, the pan-cancer mutation spectra for each gene was different to that observed in OGID, with most being nonsynonymous mutations scattered throughout the gene (Figure 5).

## Discussion

We present here the largest genetic study of overgrowth and intellectual disability performed to date, including 710 affected individuals and 636 parents. We show that OGID is a highly heterogeneous condition, involving at least 14 genes. Perturbation of epigenetic regulation is a prominent mechanism causing OGID and can be caused by mutations in at least six different genes. *NSD1* mutation is by far the most frequent cause of OGID, accounting for 240 (34%) of our series. Notably, *NSD1* is within a 2 Mb region flanked by low-copy repeats that mediate a microdeletion, which is one of the commonest causes of Sotos syndrome^16^ and was present in 29 individuals. Furthermore, exon deletions or duplications (exon CNVs) are reported in ∼5% of case subjects^17^ and were present in 9 individuals. We analyzed *NSD1* for these types of mutations, using MLPA, as they are not robustly identifiable in our exome data. We did not examine the other genes for microdeletions or exon CNVs. However, they are not known to be a major contributor to pathogenic mutations in the other genes. Even after excluding microdeletions and exon CNVs, *NSD1* is still the most common cause of OGID, accounting for 202 (28%) of our series.

The comparative analyses of genes involved in OGID with GWAS height loci and with cancer driver genes highlight intriguing similarities and differences. Our data strongly suggest that common variation impacting epigenetic regulation of gene function influences height at a population level. Further investigation of these GWAS loci would be of considerable interest, particularly in relation to advancing knowledge on how, and why, epigenetic regulation dysfunction impacts human growth.

Several genes involved in OGID are somatically mutated in a diverse range of cancers, but the spectra of mutations, particularly in the epigenetic regulation genes, is different in OGID and cancer. The underlying reasons for these differences will be complex and may include embryonic lethality of certain oncogenic mutations when they occur in the germline. Integration of germline and somatic mutational data in future research will be useful, and will likely advance functional and mechanistic understanding of the genes.

One of the most striking results of this study is the high diagnostic yield of genetic testing in OGID; a genetic cause was identified in 50% (357/710) of case subjects. This is likely to be an underestimate as we have been conservative in attributing pathogenicity to OGID gene variants and additional OGID genes almost certainly exist. Indeed, among the 132 trios in whom a definitive cause was not found, a de novo mutation possibly associated with their phenotype was present in 28; for example, two had de novo nonsynonymous variants in *XRN1*.

The diagnostic yield in our OGID series is higher than exome-sequencing studies in other phenotypes that include intellectual disability, which ranged from 13% to 35%.^22^, ^52^, ^53^, ^54^, ^55^, ^56^ The studies are not directly comparable, as most other exome studies included case subjects in which prior genetic testing was negative. Our study recruitment started prior to the discovery and clinical testing of most of the genes we report here, which allows us to provide a much better estimate of the overall contribution of rare gene mutations to this phenotype.

Given the high success rate, strong consideration should be given to using exome sequencing as a first-line diagnostic test in OGID. Height and head circumference can be easily measured and intellectual disability is readily diagnosable. Therefore, implementation of exome sequencing in OGID should be straightforward. Gene testing would provide important diagnostic and recurrence risk information to many families. Furthermore, it would increase genotype-phenotype data, which are urgently required to improve prognostic information. Of equal importance, exome sequencing in OGID would lead to the identification of new genes and new mutations in known genes. In turn, this will stimulate and facilitate scientific research, enhancing knowledge of basic biological processes controlling growth and the diverse pathologies in which human growth control is dysfunctional.

## Figures and Tables

**Table 1:** Gene and Transcript Information for 14 Genes Involved in OGID

**Gene**	**MIM Number**	**HGNC ID**	**Ensembl Transcript**	**RefSeq Transcript**
*AKT3*	611223	HGNC:393	ENST00000366539	NM_005465
*BRWD3*	300553	HGNC:17342	ENST00000373275	NM_153252
*CHD8*	610528	HGNC:20153	ENST00000399982	NM_001170629
*DNMT3A*	602769	HGNC:2978	ENST00000264709	NM_175629
*EED*	605984	HGNC:3188	ENST00000263360	NM_003797
*EZH2*	601573	HGNC:3527	ENST00000320356	NM_004456
*GPC3*	300037	HGNC:4451	ENST00000370818	NM_004484
*HIST1H1E*	142220	HGNC:4718	ENST00000304218	NM_005321
*MTOR*	601231	HGNC:3942	ENST00000361445	NM_004958
*NFIX*	164005	HGNC:7788	ENST00000360105	NM_002501
*NSD1*	606681	HGNC:14234	ENST00000439151	NM_022455
*PIK3CA*	171834	HGNC:8975	ENST00000263967	NM_006218
*PPP2R5D*	601646	HGNC:9312	ENST00000485511	NM_006245
*PTEN*	601728	HGNC:9588	ENST00000371953	NM_000314

## Web Resources

ExAC Browser, http://exac.broadinstitute.org/
ExPASy Translate Tool, http://web.expasy.org/translate/
g:Profiler, http://biit.cs.ut.ee/gprofiler/
GenBank, http://www.ncbi.nlm.nih.gov/genbank/
GeneReviews, Eng, C. (1993). PTEN hamartoma tumor syndrome. https://www.ncbi.nlm.nih.gov/books/NBK1488/
GeneReviews, Tatton-Brown, K., Cole, T.R.P., and Rahman, N. (1993). Sotos syndrome. https://www.ncbi.nlm.nih.gov/books/NBK1479/
ICR1000 UK Exome Series, http://www.icr.ac.uk/icr1000exomes
OMIM, http://www.omim.org/
OpEx NGS Pipeline, http://www.icr.ac.uk/opex
Picard, http://broadinstitute.github.io/picard/
Protein calculator, http://pepcalc.com/protein-calculator.php
TumorPortal, http://www.tumorportal.org/

## Supplemental Data

Document S1. Figure S1 and Supplemental Note

Table S1. Summary Molecular and Phenotypic Data from 710 Individuals with OGID

Document S2. Article plus Supplemental Data
