# Supplementary material for: Mutations in Epigenetic Regulation Genes Are a Major Cause of Overgrowth with Intellectual Disability
Source: Am J Hum Genet. 2017 May 4;100(5):725–36. doi: 10.1016/j.ajhg.2017.03.010 (PMC5420355; doi:10.1016/j.ajhg.2017.03.010)
Supplement: Document S1. Figure S1 and Supplemental Note [file mmc1.pdf]

The American Journal of Human Genetics, Volume *100*

Supplemental Data

Mutations in Epigenetic Regulation Genes Are a Major Cause of Overgrowth with Intellectual Disability

Katrina Tatton-Brown, Chey Loveday, Shawn Yost, Matthew Clarke, Emma Ramsay, Anna Zachariou, Anna Elliott, Harriet Wylie, Anna Ardissone, Olaf Rittinger, Fiona Stewart, I. Karen Temple, Trevor Cole, Childhood Overgrowth Collaboration, Shazia Mahamdallie, Sheila Seal, Elise Ruark, and Nazneen Rahman

Figure S1. Height GWAS regional association plots for OGID genes

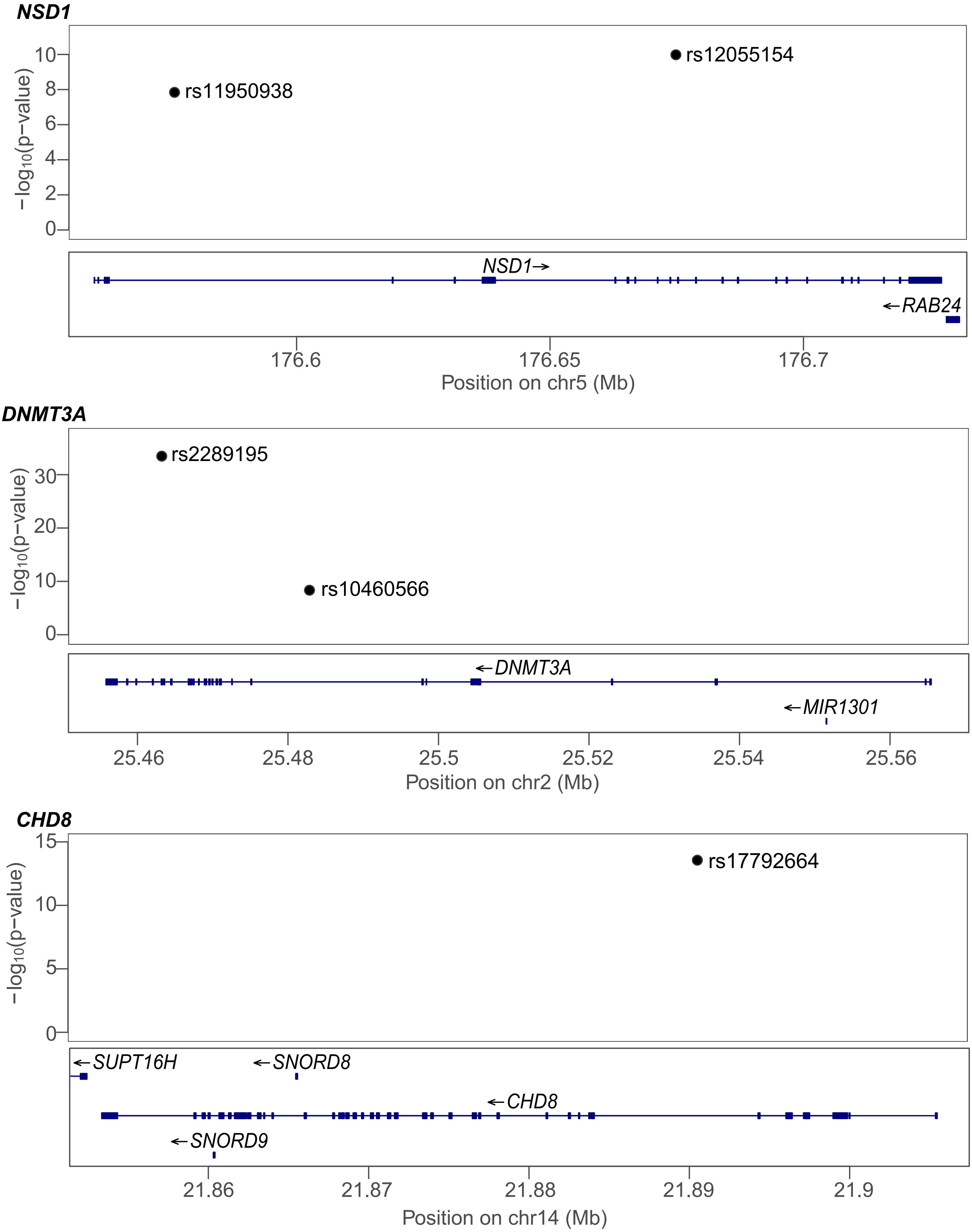

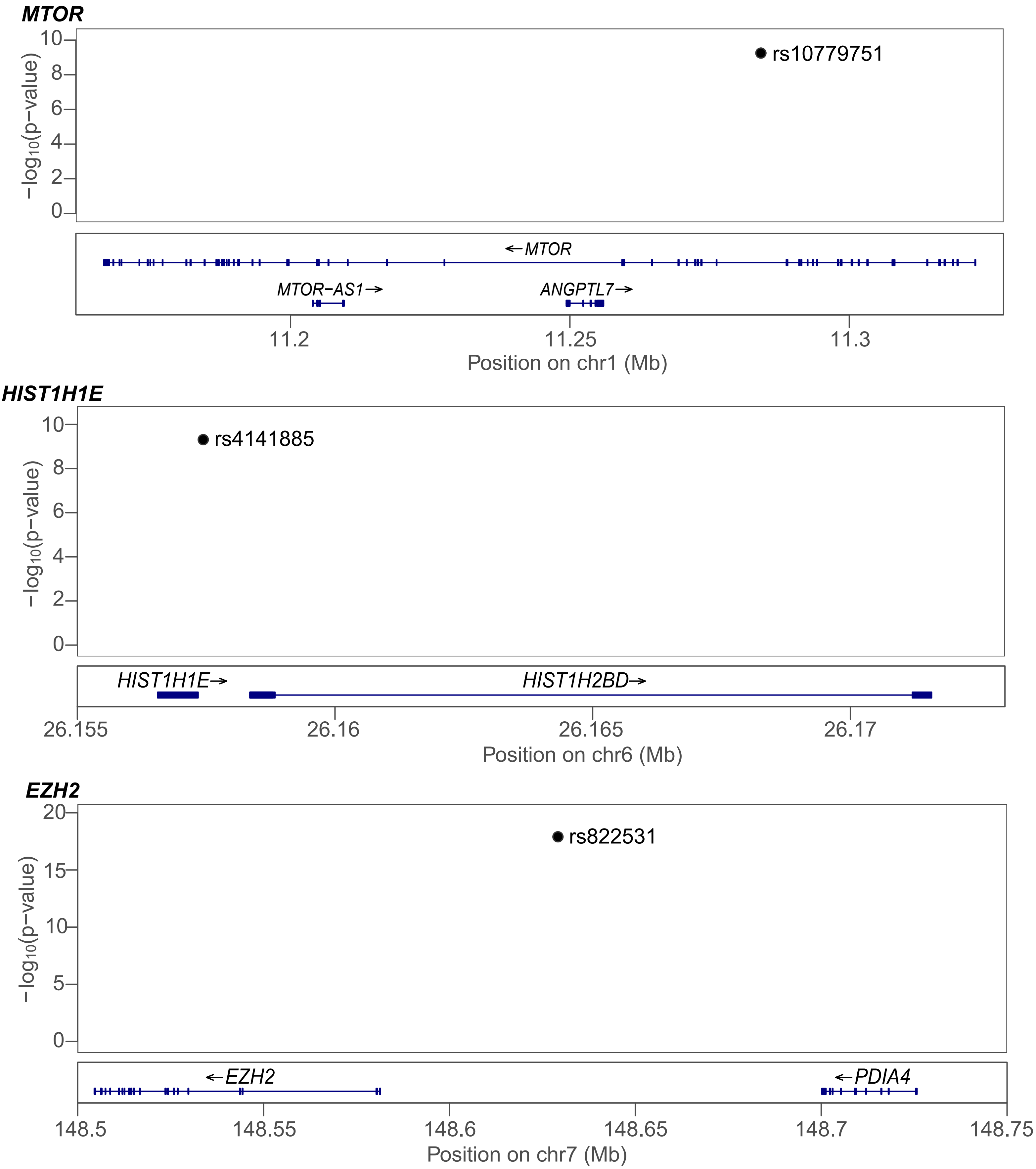

Regional association plots showing variants with significant associations in the height GWAS study by Wood et al.[27] that are in the vicinity of OGID genes.

Supplemental Note. The Childhood Overgrowth Collaboration

The following individuals coordinated recruitment and collection of the families and samples.

M-C. Addor, M. Akgul, L. Aksglaede, M. Ahmed, D. Amor, K. Anderson, R. Anderson, S. Andries, H. Archer, R. Armstrong, P. Ashton-Prolla, M. Bahceci, M. Balasubramanian, D. Baralle, D. Barge, A. Barnicoat, M. Barrow, J. Barwell, G. Baujat, G. Baynam, P. Beales, K. Becker, E. Beckh-Arnold, A. Ben-Yehuda, J. Berg, B. Bernhard, S. Bhal, M. Bhat, J. Birch, L. Bird, M. Bitner-Glindzicz, E. Blair, J. Bliek, M. Blyth, A. Bottani, M. Bouma, M. Boxill, F. L. Bradley, A. Brady, Breatnach, G. Brice, B. Buehler, A. Burke, J. Burn, J. Campbell, N. Canham, B. Castle, K. Chandler, R. Chandrasena, E. Chang, C. Christenden, C. Chu, D. Cilliers, A. Clarke, J. Clayton-Smith, C. Clericuzio, V. Clowes, T. Cole, A. Colley, A. Collins, F. Connell, J. Cook, I. Cordeiro, E. Crocker, Y. Crow, V. Culic, T. Cushing, T. Dabir, A. Dalton, S. Danda, R. Davidson, S. Davies, R. Day, D. Dearnaley, M-A. Delrue, M. De Roy, V. de Soberanis, M. de Ville, N. Dennis, C. Deshpande, B. Desouza, L. Devlin, A A. Dieckmann, -M. Differ, R. Dinwiddie, A. Dixit, A. Dobbie, J. Dominguez, A. Donaldson, D. Donnai, D. Donnelly, H. Dorkins, M. Doz, J. Dupont, D. Eastwood, M. Edwards, I. Ellis, F. Elmslie, L. Escobar, R. Evans, F. Faravelli, C. Fauth, H. Firth, R. Fisher, T. Fiskerstrand, D. Fitzpatrick, A. Flanagan, F. Flinter, P. Foley, A. Foster, N. Foulds, W. Foulkes, J. Franklin, A. Fryer, H. Fryssira, A. Gallagher, S. Garcia, C. Gardiner, M. Gardner, C. Garrett, B. Gener, M. Gerrard, R. Gibbons, Y. Gillerot, H. Goel, D. Goudie, K. Gowrishankar, C. Graham, A. Green, N. Gregersen, J. Hale, M. Hamilton, J. Harper, R. Harrison, V. Harrison, A. Henderson, P. Henman, R. Hennekam, E. Hobson, S. Hodgson, M. Holder, S. Holder, T. Homfray, D. Horovitz, H. Hughes, Z. Huma, M. Hunter, J. Hurst, W-L. Hwu, A. Irvine, M. Irving, L. Izatt, M-L. Jacquemont, S. Jagadeesh, L. Jenkins, U. Jensen, C. Jessen, D. Johnson, J. Johnson, E. Jones, L. Jones, A. Jorgensen, D. Josifova, S. Joss, Dr. Kanabar, P. Kannu, K. Keppler-Noreuil, B. Kerr, H. Kingston, J. Kingston, U. Kini, E. Kinning, A. Krause, V. Krishnamurthy, A. Kumar, D. Kumar, A. Medeira, V. Meiner, C. Mercer, K. Milstein, Y. Miyoshi, E. Moran, K. Lachlan, W. Lam, P. Lapunzina, M. Lees, N. Leonard, G. Levitt, I. Lewis, J. Liebelt, A. Livesey, C. Longman, T. Lopponen, Dr Lozano, A. Lucassen, P. Lunt, S-A Lynch, S. Lyonnet, J. MacDonnell, A. Magee, E. Maher, S. Maitz, A. Male, S. Mansour, C. Marcelis, E. McCann, V. McConnell, T. McDevitt, M. McEntagart, J. McGaughran, G. McGillivray, R. McGowan, S. McKee, C. McKeown, C. Meany, S. Mehta, K. Metcalfe, Z. Miedzybrodzka, S. Mohammed, G. Monaghan, T. Montgomery, A. Morgan, B. Morland, P. Morrison , J. Morton, R. Mudgal, A. Munaza, V. Murday, S. Nampoothiri, K. Nathanson, K. Neas, A. Nemeth, G. Neri, R. Newbury-Ecob, C. Nur Semerci, C. Ockeloen, C. Oley, C. Owen, K. Ozono, Panarello, S-M. Park, M. Parker, C. Patel, M. Patton, S. Payne, M. Pearson, J. Piard, D. Pilz, M. Pinkney, B. Plecko, M. Pocha, G. Poke, R. Posmyk, C. Pottinger, K. Prescott, S. Price, K. Pritchard-Jones, A. Proctor, V. Puthi, O. Quarrell, A. Raas-Rothchild, E. Rahikkala, W. Raith, J. Rankin, L. Raymond, G. Rea, L. Read, W. Reardon, E. Reid, H.Rees, N. Revencu, O. Rittinger, M. Robards, A. Roposch, E. Rosser, D. Rourke, D. Ruddy, A. Saggar, N. Saleh, V. Saletti, J. Sampson, R. Sandford, H. Santos, A. Sarkar, R. Scott, I. Scurr, C. Searle, A. Selicorni, R. Semple, S. Sharif, A. Shaw, C. Shaw-Smith, D. Shears, J. Shelagh, N. Shur, L. Side, M. Simon, F. Skovby, G. Smith, S. Smithson, M. Splitt, M. Stevens, A. Stewart, F. Stewart, H. Stewart, K. Stopps, C. Stumpel, K. Stuurman, D. Subramanian, M. Suri, A. Swain, E. Sweeney, K. Szakszon, Y. Sznajer, G. Tanateles, A. Taylor, C. Taylor, M. Teixeira, I.K. Temple, E. Thomas, E. Thompson, F. Thonney, M. Tischowitz, J. Tolmie, S. Tomkins, S. Turkmen, A. Turner, P. Turnpenny, M. Van-Haelst, L. Van Maldergem, P. Vasudevan, I. Veenstra-Knol, C. Verellen, I.C. Verma, J. Vigneron, E. Wakeling, L. Wainwright L. Walker, D. Weaver, P. Wheeler, K. White, S. White, M. Whiteford, D. Williams, L. Wilson, R. Winter, G. Woods, M. Wright, N. Yachelevich, A. Yeung, A. Zankl
